# In a quest for better outcome prediction in cardiogenic shock

**DOI:** 10.1002/ehf2.15224

**Published:** 2025-01-21

**Authors:** Wiktor Kuliczkowski

Improving outcomes in cardiogenic shock seems to be currently the holy grail of modern cardiology. Intravenous agents have their shortcomings, as well as mechanical circulatory support.^1^, ^2^, ^3^, ^4^ Improving outcomes could start with better outcome prediction, which could aim our efforts at populations who are at higher risks.^5^ It is quite well recognized that cardiogenic shock is ‘purely cardiogenic’ up to 12–24 h from its start, later changing into multiorgan failure which in the end is the major direct cause of death. This is exactly what SCAI classification shows—cardiogenic shock as a continuity, where a change from SCAI C do D or E poses a higher risk for death, while the opposite direction is connected with a better outcome.^6^ Is SCAI classification the best we have? It was shown that depending on the investigator, the same SCAI stage can end up with a different death rate, so there is still some room for improvement.^7^

The first organs apart from the heart, which suffers quite early from hypoperfusion, are the kidneys. Kidney function is included in SCAI staging, but its more detailed classification brings KDIGO criteria for acute kidney injury. In the current issue of the *ESC Heart Failure* journal, Li et al. show the results of their retrospective analysis of patients with cardiogenic shock, mainly post‐cardiotomy, who needed VA ECMO (spell out) for their stabilization. The authors checked if there would be any improvement in the prediction of in‐hospital mortality after adding to SCAI shock stage AKI stage, serum lactate level, SOFA score, SVAE score and VIS score as all of the proposed markers/scores have already been shown to impact the prognosis IN heart failure.^8^, ^9^ The best and additional effect was obtained with AKI stage, which together with SCAI sock stage gave the area under the receiver operating characteristic curve of 0.754 (95% confidence interval: 0.690 to 0.811). When we obtain the area under the ROC close to or above 0.80, statisticians say it is a fair effect, so we can agree that the AKI and SCAI combination worked well in this population. There are some drawbacks of the paper: first, it is mainly obtained in a quite rare subgroup of patients with cardiogenic shock post cardiotomy. We now know that clinical course and outcome differ depending on more common cardiogenic shock aetiology like myocardial infarction or decompensated heart failure,^10^, ^11^ and it seems quite difficult to extrapolate presented results to those aetiologies, although authors try to do it. Second, VA ECMO was mainly peripheral, but LV unloading was used only in 1.9% of patients and with IABP, which acts differently in this setting.^12^ LV unloading seems crucial in VA ECMO use,^13^ but more potent microaxial flow pumps were not available at that time, and it also renders it difficult to match the studied group to current daily practice, where LV unloading with microaxial flow pump seems to impact mortality.^14^, ^15^, ^16^ And finally, due to a small number of patients in AKI and SCAI subgroups, the authors obtained relatively wide confidence intervals in their multivariate analysis, which poses caution on this part of the results. Nevertheless, I would like to congratulate Li et al. for their meticulous work and presented results as they add useful information into field of cardiogenic shock treatment.

Now, the question arises of how we can use the data from currently published study. As the AKI is so frequent in cardiogenic shock and adds to death prediction, it would be wise to start a trial where CRRT is started earlier in cardiogenic shock not waiting for high grade of AKI to develop. Maybe changing from heart‐centric view to multiorgan‐centric view in cardiogenic shock treatment with early support not only of the heart or lungs with mechanical devices but also kidneys (which seems to be the third crucial organ in this matter) would result in better outcomes achievement in the future.^17^, ^18^, ^19^, ^20^
